# Invisible burdens: reframing maternal mental health as a core priority in conflict-affected and low-resource settings

**DOI:** 10.3389/fgwh.2026.1989039

**Published:** 2026-10-13

**Authors:** Ilyas Abdullahi Khalif, Yusuf Abdullahi Hubow, Ahmed Mohamed Omar, Nour Ahmed Dahir, Aniso Mohamed Abdi

**Affiliations:** Department of Public Health, Faculty of Medicine and Health Sciences, Hormuud University, Mogadishu, Somalia

**Keywords:** conflict-affected settings, low- and middle-income countries, maternal mental health, perinatal mental health, women's health equity

## Abstract

Maternal mental health remains an overlooked dimension of global women's health, particularly in conflict-affected and low-resource settings where psychological distress is intensified by poverty, displacement, violence, gender inequality, and fragile health systems. Although maternal health agendas have achieved progress in reducing mortality, survival-focused approaches often fail to capture the psychological burdens that shape women's well-being, caregiving capacity, and intergenerational outcomes. This Perspective argues that maternal mental health should be reframed as a core component of maternal health rather than a secondary concern. Drawing on a life-course and gender-responsive perspective, we propose a conceptual framework linking conflict and structural inequality with social determinants, maternal psychological outcomes, and consequences for mothers and children. We highlight the need for integrated health-system responses that combine mental health screening, psychosocial support, community-based care, and gender-responsive policies to achieve equitable and sustainable improvements in women's health.

## 1 Introduction

Global maternal health has achieved important gains through reduced maternal mortality and expanded access to reproductive and maternity care, yet progress remains incomplete and deeply unequal (1, 2). A survival-focused agenda is too narrow: contemporary maternal health scholarship argues that well-being, dignity, rights, and person-centred care are integral to maternal health, while decades of global prioritization have often privileged biomedical indicators over the social and political determinants that shape women's lives (3, 4).

Within this gap, maternal depression, anxiety, psychological distress, and trauma remain systematically under-recognised, especially in low-resource settings where roughly one in four women experience perinatal depression and one in five experience anxiety disorders (5). This burden is intensified by poverty, gender inequality, violence, food insecurity, weak service availability, and stigma (6, 7). In conflict-affected and displaced populations, pre-migration trauma, ongoing deprivation, unstable housing, and disrupted care further elevate risks of depression, anxiety, post-traumatic stress disorder (PTSD) and stress (8). Yet maternal mental health remains conspicuously absent from many maternal, newborn, child, and adolescent health (MNCAH) framework and insufficiently translated into policy and scaled programming (9). This Perspective therefore argues that maternal mental health must be reframed as a core global women's health priority in conflict-affected and low-resource settings, and advances a critical agenda for rethinking how policy, research, and health systems define maternal health itself.

## 2 Maternal mental health vulnerability in conflict settings

Conflict and fragility create distinct maternal mental health vulnerabilities because pregnancy and the postpartum period unfold within overlapping conditions of threat, loss, and deprivation rather than as isolated clinical events. Armed violence, forced displacement, chronic insecurity, and poverty intensify anxiety, perceived stress, and traumatic stress through cumulative pathways that begin before migration, continue during displacement, and often persist long after fighting subsides (10, 11). Among migrant women, one in four experience perinatal depression, one in five anxiety, and forced migrants show a particularly high burden of PTSD, while post-political conflict settings report similarly elevated rates of anxiety, depression, and PTSD (11). These risks are not explained by trauma exposure alone: social isolation, discrimination, poor partner support, housing precarity, food insecurity, and disrupted access to prenatal and postnatal care compound distress and can mediate the mental health effects of violence and displacement (10).

Pregnant and postpartum women are especially vulnerable because this period already involves major biological adjustment, heightened emotional demands, and immediate responsibility for infant survival and caregiving (8). In conflict settings, these normative transitions are overlaid by trauma, family separation, and the erosion of social support structures that ordinarily buffer stress (12). Maternal psychological distress extends beyond the woman herself and may affect children across the life course. Prenatal stress is associated with fetal brain changes and poorer infant cognitive and social-emotional outcomes, while perinatal distress can impair mother–infant bonding, breastfeeding, caregiving quality, and early child development, with consequences for family functioning and intergenerational wellbeing (13, 14). These effects may persist beyond infancy: evidence from conflict-affected settings indicates that maternal depressive symptoms and exposure to traumatic events are associated with poorer early childhood mental health, while child emotional and behavioral difficulties may, in turn, exacerbate maternal psychological distress, creating a potentially recursive cycle of adversity (15). In conflict-affected and low-resource settings, these pathways may be amplified by displacement, poverty, food insecurity, family separation, and disrupted health and social services. Maternal mental health should therefore be understood not only as an individual clinical concern but also as an important determinant of child wellbeing, family functioning, and health across generations (15, 16).

## 3 Structural neglect of maternal mental health

This section examines mortality-centred metrics, service fragmentation, and humanitarian triage as interconnected reasons maternal mental health remains peripheral in global women's health agendas.

Contemporary maternal health policy still privileges survival over wellbeing. Global action has largely concentrated on the leading biomedical causes of death, while giving less attention to the underlying determinants of maternal health and to women's broader wellbeing and quality of life (3). This bias is also visible in measurement: although perinatal mental health disorders contribute to both maternal morbidity and mortality, there are still no global indicators routinely used to track perinatal mental health status or progress, which reinforces their policy invisibility (17). The result is a narrowed model of maternal health in which psychosocial suffering is treated as secondary, despite evidence that psychological conditions are major contributors to pregnancy-related death and are closely linked to adverse obstetric, infant, and intergenerational outcomes (12).

The marginal status of maternal mental health is further entrenched by fragmented systems of care. In many low- and middle-income settings, maternal and mental health services remain organized in silos, with weak policy support, limited workforce capacity, absent clinical guidelines, and referral systems that fail to identify or follow women with psychological distress (18). More broadly, LMIC health services are often disease-specific and poorly integrated, with financing, supervision, and infrastructure deficits that undermine people-centred continuity of care (19). Such discontinuities are not merely operational flaws; they are structural inequities that reduce effective maternal care coverage and disproportionately affect marginalized women (20).

In humanitarian settings, this structural weakness is magnified by emergency logics that prioritize food, shelter, infection control, and obstetric survival, while psychosocial care remains limited, delayed, or externalized. Conflict, displacement, and public health emergencies intensify maternal vulnerability and disrupt already fragile systems (3). Yet conflict-affected and displaced women remain underserved in psychosocial and social protection services, even as distress, PTSD, anxiety, and parenting stress rise under conditions of violence, insecurity, and loss (21). Maternal mental health neglect should therefore be understood not as a peripheral omission, but as a structural weakness in global women's health systems: a sign that health agendas still respond more readily to visible threats to life than to the less visible conditions that shape women's capacity to survive, function, parent, and recover (3).

## 4 Reframing maternal mental health

Maternal health requires a shift from a survival-focused biomedical model to a biopsychosocial and life-course model that recognizes mental health as intrinsic to women's health, not ancillary to it.

An exclusive focus on mortality and obstetric complications is now too narrow for maternal health policy, because surviving pregnancy is not equivalent to achieving maternal health and wellbeing across the life course (3). Contemporary maternal health scholarship increasingly frames maternal ill-health as the product of interacting social, biological, and health-system determinants, making a solely biomedical lens analytically and programmatically insufficient (3). Within this broader view, maternal mental health is a fundamental dimension of women's health because psychological distress shapes care use, social functioning, and outcomes for mothers, infants, and families across pregnancy and the postpartum period (22).

Integrating psychological well-being into maternal health definitions is therefore conceptually necessary, not merely clinically desirable. Biopsychosocial models show that perinatal mental health emerges through cross-system interactions among neuroendocrine, immune, nutritional, psychological, relational, and social processes, and that these pathways can carry intergenerational effects (23). Research and clinical frameworks accordingly argue for holistic, person-centred care that combines screening, psychosocial support, and context-sensitive responses rather than treating mental disorders as isolated complications (24).

A gender-responsive orientation is central to this reframing because maternal health inequities are produced through power relations, constrained agency, violence, limited resources, and exclusion from decision-making (4). Interventions that engage social relationships and gender dynamics can improve both mental health and care-seeking, as shown by integrated and gender-synchronised models in LMICs (25). Framing maternal mental health in this way aligns directly with the Sustainable Development Goals (SDGs), particularly SDG 3, which addresses health and well-being, and SDG 5, which addresses gender equality, while offering the conceptual basis for a framework that links clinical care, social protection, and rights-based health-system design (3).

## 5 Proposed framework: linking conflict, social determinants, and maternal mental health

Figure 1 conceptualizes maternal mental health not as an isolated clinical condition, but as the psychological expression of conflict layered onto pre-existing structural inequality. Conflict erodes the political, economic, and health system conditions that shape everyday survival, while structural inequities distribute that harm unevenly across women already marginalized by poverty, low education, insecure housing, and weak service access (26). In this framework, conflict operates as a structural determinant that intensifies adverse social exposures rather than a discrete shock alone, producing cascades of deprivation, insecurity, and exclusion across the perinatal period (27).

**Figure 1:**
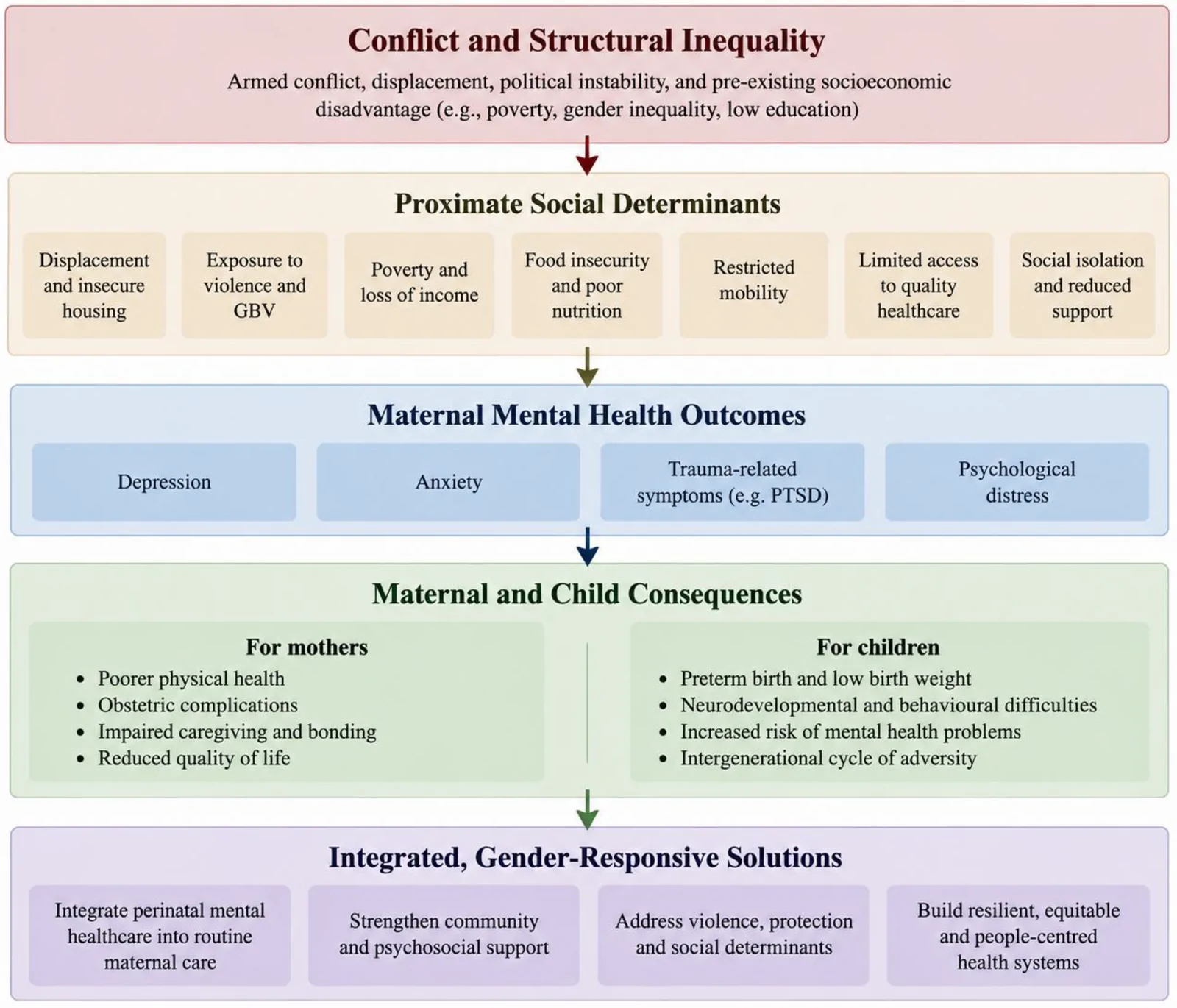
Proposed framework: linking conflict, social determinants, and maternal mental health.

These structural disruptions become biologically and socially consequential through proximate determinants: displacement, violence, gender-based violence (GBV), food insecurity, restricted mobility, loss of income, and breakdown of care infrastructures. In Gaza, most pregnant women reported very low income, inadequate food quantity or diversity, limited antenatal care, and high stress, with transport costs a major barrier to care (28). Across fragile settings, fear, trauma, poverty, gender norms, and service collapse interact in mutually reinforcing ways that suppress help-seeking and deepen vulnerability (29).

The next step in the pathway is the accumulation of maternal depression, anxiety, trauma responses, and psychological distress. These outcomes are not merely co-travelers of adversity; they are mediating mechanisms through which conflict becomes embodied. Conflict violence and deprivation have been linked to perceived stress, generalized anxiety, and altered stress physiology after childbirth, while women in post-conflict primary care settings show high prevalence of depressive and anxiety symptoms, supporting the argument that maternal mental health should be treated as a core outcome of humanitarian and health-system failure (30).

The final pathway runs from maternal mental health to intergenerational consequence. Maternal depressive symptoms are longitudinally associated with child mental health in conflict-affected settings, and child difficulties can, in turn, exacerbate later maternal depression, indicating a recursive rather than linear burden (15). War-related prenatal stress is also associated with low birth weight, prematurity, miscarriage, and later neurodevelopmental risk, with maternal trauma responses shaping children's emotional and behavioral difficulties (16). The framework therefore argues that effective response must interrupt the pathway at multiple levels through integrated perinatal mental healthcare, community and psychosocial support, and gender-transformative, multisectoral policy that addresses violence, social support, and economic precarity alongside clinical need (31).

The framework illustrates that maternal mental health outcomes in conflict-affected and low-resource settings emerge through interconnected pathways rather than isolated clinical mechanisms. Conflict and structural inequalities create social and environmental exposures that increase psychological vulnerability, while maternal distress contributes to consequences extending beyond the individual woman to children and future generations. The framework highlights the importance of multi-level responses that combine integrated healthcare, psychosocial support, gender-responsive policies, and social protection interventions.

## 6 Practical priorities for integrating maternal mental health

Maternal mental health screening should be embedded into routine antenatal and postnatal contacts, but only where screening is linked to clinical assessment, referral, and follow-up. In conflict-affected settings, implementation should prioritize brief, validated screening approaches that can be incorporated into existing maternal and child health contacts without creating parallel services. Women who screen positive should have access to a defined stepped-care pathway, beginning with appropriate psychosocial support and progressing to specialist assessment for severe, persistent, or high-risk conditions. Where specialist services are scarce, referral pathways should identify available district, hospital, humanitarian, or remote mental health resources and clarify responsibilities for follow-up. Evidence supports integrating screening into maternal and child health platforms because it improves access and can reduce stigma, yet implementation in low- and middle-income countries remains constrained by weak referral pathways and limited evidence on large-scale delivery (32). Programmatic experience from Sri Lanka further suggests that two-stage screening, combining brief tools with clinical interview, can identify women needing support while reducing unnecessary pressure on specialist services (33).

A realistic delivery model in low- and middle-income countries is task-shared, culturally adapted psychosocial care delivered by trained midwives, nurses, community health workers, or peers, with competency-based training, supportive supervision, and clear escalation pathways for severe illness (34). In conflict-affected settings, implementation should include refresher training, regular supervisory support, simple referral protocols, and mechanisms for consultation when non-specialist providers encounter complex cases. Where multiple organizations provide services, coordination among government, humanitarian, and nongovernmental providers is also important to clarify referral responsibilities and reduce gaps in follow-up. These approaches can improve access, but sustainability remains threatened by intermittent funding, workforce turnover, and uneven resource distribution (35).

Continuity of care is particularly important when conflict disrupts health facilities, displaces communities, or restricts movement. Maternal mental health services should therefore be incorporated into existing antenatal, postnatal, primary healthcare, and humanitarian service platforms rather than relying exclusively on fixed specialist facilities. Where facility access is interrupted, community health workers, outreach teams, home visits, peer-support groups, and other locally adapted delivery mechanisms can provide basic psychosocial support and facilitate referral to higher levels of care (31). Such approaches should be adapted to local safety conditions, privacy constraints, language, cultural understandings of distress, and the availability of referral services. Community-based delivery should complement rather than replace facility-based care, particularly for women requiring clinical assessment or specialist intervention.

Humanitarian responses should also include maternal mental health indicators, since current measurement systems rarely capture screening, service access, referral completion, follow-up, or provider training, and most existing indicators were developed outside low- and middle-income country contexts (17). A feasible monitoring approach could track the proportion of eligible women screened, the proportion requiring further assessment, completion of referrals, follow-up attendance, and provider training and supervision coverage. These indicators should be incorporated into existing health information and humanitarian monitoring systems where feasible rather than creating parallel reporting structures. Future research should prioritize pragmatic trials, implementation and cost-effectiveness studies, longitudinal designs, and context-valid measures in conflict settings, where evidence remains sparse despite high and persistent risk (32). Research should assess not only clinical effectiveness but also feasibility, acceptability, equity, safety, continuity, and sustainability under conditions of insecurity and service disruption. Addressing these interconnected challenges requires coordinated health-system strategies that move beyond isolated interventions toward sustainable integration of maternal mental health within routine maternal and humanitarian care. The following table summarizes the major implementation barriers and evidence-based actions required to strengthen maternal mental health services in conflict-affected and low-resource settings.

The barriers summarized in Table 1 highlight that maternal mental health integration requires coordinated responses across policy, workforce capacity, cultural adaptation, and service delivery. Addressing these interconnected challenges is essential for establishing sustainable and equitable maternal mental health systems in fragile and low-resource settings.

**Table 1:** Priority barriers and evidence-based health-system actions for integrating maternal mental health into conflict-affected and low-resource settings.

Major barrier	Consequences for women's health	Evidence-based priority action	Citations
Weak policy prioritization and financing for maternal mental health	Low prioritization and inadequate resource allocation limit routine identification and treatment of maternal mental health conditions, delaying care and reinforcing inequitable access.	Integrate maternal mental health into national maternal and primary healthcare policies, establish dedicated financing mechanisms, and implement routine antenatal and postnatal mental health screening with appropriate tools and budget allocation.	(36, 37)
Shortage of trained and supported frontline providers	Limited provider knowledge and confidence reduce recognition of maternal mental health symptoms, weaken assessment, and contribute to delayed or missed care among pregnant and postpartum women.	Implement task-sharing approaches involving trained midwives, nurses, community health workers, and other non-specialist providers supported by structured training, supervision, and monitoring systems.	(37, 38)
Absent or fragmented screening, referral, and stepped-care pathways	Women are frequently identified late or only after symptoms become severe because referral systems, follow-up mechanisms, and specialist linkages remain unclear.	Establish standardized screening, referral, follow-up, and escalation pathways integrated into routine maternal healthcare platforms and connected with specialist mental health services when required.	(36, 39)
Stigma, cultural mismatch, and low help-seeking demand	Stigma, communication barriers, and culturally inappropriate models discourage disclosure and care-seeking, increasing delays and leaving psychological distress untreated.	Develop culturally adapted interventions through community engagement, use locally meaningful understandings of distress, and combine facility-based care with awareness and social support strategies.	(34, 36)
Fragile, under-resourced, and fragmented service platforms in humanitarian and low-resource settings	Conflict and health-system fragility disrupt facilities, workforce capacity, privacy, continuity of care, and access to sustained maternal mental health services.	Integrate brief psychosocial interventions into existing maternal and primary healthcare platforms, strengthen coordination between humanitarian and government services, and expand feasible community-based delivery models.	(34, 39)

This table synthesizes evidence-based barriers and health-system actions for integrating maternal mental health into routine maternal healthcare and humanitarian responses in low-resource and conflict-affected settings.

## 7 Conclusion

Maternal mental health remains an invisible burden because it is consistently overshadowed by more visible obstetric outcomes, underfunded in policy and practice, and further concealed by stigma, silence, and weak routine detection within maternal health systems. In low-resource settings, this neglect is especially consequential because the treatment gap is vast, access to appropriate care remains limited, and maternal mental health evidence is still insufficiently translated into policy, programming, and scalable service delivery.

Conflict and poverty intensify this burden by concentrating structural adversity in women's lives, including displacement, gender inequality, violence, food insecurity, weak social protection, and fragile health systems. These conditions do not simply increase psychological distress; they expose the limits of maternal health models that define progress too narrowly through survival, service contact, or biomedical risk alone. A women's health agenda that does not account for mental well-being cannot fully address dignity, agency, quality of care, or equity across pregnancy, childbirth, and the postpartum period.

Reframing maternal mental health as core, rather than peripheral, is therefore essential to sustainable development: psychological well-being is inseparable from maternal dignity, family functioning, child outcomes, and women's ability to live healthy lives of value. The way forward is not a parallel mental health agenda, but integrated, gender-responsive, intersectional, and life-course approaches that embed maternal mental health within primary care, social policy, and broader systems of women's health and rights.

## Data Availability

The original contributions presented in the study are included in the article/supplementary material, further inquiries can be directed to the corresponding author.

## References

[1] Solnes Miltenburg A Kvernflaten B Meguid T Sundby J. Towards renewed commitment to prevent maternal mortality and morbidity: learning from 30 years of maternal health priorities. Sex Reprod Health Matters. (2023) 31:2174245. 10.1080/26410397.2023.2174245 36857112 PMC9980022

[2] Myatra SN Tripathy S Einav S. Global health inequality and women—beyond maternal health. Anaesthesia. (2021) 76:6–9. 10.1111/anae.15431 33682098

[3] Souza JP Day LT Rezende-Gomes AC Zhang J Mori R Baguiya A. A global analysis of the determinants of maternal health and transitions in maternal mortality. Lancet Glob Health. (2024) 12:e306–16. 10.1016/S2214-109X(23)00468-0 38070536

[4] Bohren MA Iyer A Barros AJD Williams CR Hazfiarini A Arroyave L. Towards a better tomorrow: addressing intersectional gender power relations to eradicate inequities in maternal health. eClinicalMed. (2024) 67:102180. 10.1016/j.eclinm.2023.102180 PMC10837533 38314054

[5] Roddy Mitchell A Gordon H Lindquist A Walker SP Homer CSE Middleton A. Prevalence of perinatal depression in low- and middle-income countries: a systematic review and meta-analysis. JAMA Psychiatry. (2023) 80:425–31. 10.1001/jamapsychiatry.2023.0069 36884232 PMC9996459

[6] Roddy Mitchell A Gordon H Atkinson J Lindquist A Walker SP Middleton A. Prevalence of perinatal anxiety and related disorders in low- and middle-income countries: a systematic review and meta-analysis. JAMA Netw Open. (2023) 6:e2343711. 10.1001/jamanetworkopen.2023.43711 37976063 PMC10656650

[7] Pan T Zeng Y Chai X Wen Z Tan X Sun M. Global prevalence of perinatal depression and its determinants among rural women: a systematic review and meta-analysis. Depress Anxiety. (2024) 2024:1882604. 10.1155/2024/1882604 40226692 PMC11919136

[8] Almeida LM Moutinho AR Siciliano F Leite J Caldas JP. Maternal mental health in refugees and migrants: a comprehensive systematic review. J Int Migr Integr. (2024) 25:209–22. 10.1007/s12134-023-01071-3

[9] Abajobir A Sidze EM Wainaina C Gerbaba MJ Wekesah FM. The epidemiology of maternal mental health in Africa: a systematic review. Arch Womens Ment Health. (2025) 28:997–1089. 10.1007/s00737-025-01563-4 40220206 PMC12436556

[10] Koning SM Adam EK Kapoor A McDade TW. Echoes of conflict and displacement in maternal health: life-course violence, timing, and maternal stress after childbirth at the northern Thailand-Myanmar border. Psychoneuroendocrinology. (2025) 171:107189. 10.1016/j.psyneuen.2024.107189 39378689 PMC11895776

[11] Stevenson K Fellmeth G Edwards S Calvert C Bennett P Campbell OMR. The global burden of perinatal common mental health disorders and substance use among migrant women: a systematic review and meta-analysis. Lancet Public Health. (2023) 8:e203–16. 10.1016/S2468-2667(22)00342-5 36841561

[12] Ether ST Afrin S Habib NN Akter F Chowdhury AT Sayeed A. Managing pre and postpartum mental health issues of refugee women from fragile and conflict-affected countries: a systematic review. Public Health in Pract. (2025) 9:100573. 10.1016/j.puhip.2024.100573 PMC11743345 39835221

[13] Wu Y Espinosa KM Barnett SD Kapse A Quistorff JL Lopez C. Association of elevated maternal psychological distress, altered fetal brain, and offspring cognitive and social-emotional outcomes at 18 months. JAMA Netw Open. (2022) 5:E229244. 10.1001/jamanetworkopen.2022.9244 35486403 PMC9055453

[14] Basile-Ibrahim B Combellick J Mead TL Sorensen A Batten J Schafer R. The social context of pregnancy, respectful maternity care, biomarkers of weathering, and postpartum mental health inequities: a scoping review. Int J Environ Res Public Health. (2024) 21:480. 10.3390/ijerph21040480 38673391 PMC11049830

[15] Rees SJ Mohsin M Klein L Steel Z Tol W Dadds M. The impact of maternal depressive symptoms and traumatic events on early childhood mental health in conflict-affected Timor-Leste. BJPsych Open. (2022) 8:e51. 10.1192/bjo.2022.20 35197139 PMC8935917

[16] Riquelme-Gallego B Ramos-Soberbio L Leno-Duran E Martínez-Vázquez S Caparros-Gonzalez RA. Adverse fetal and neonatal impact of war conflicts during pregnancy: a systematic review. IUBMB Life. (2025) 77:e70006. 10.1002/iub.70006 39981676

[17] Layton E Roddy Mitchell A Kennedy E Moran AC Palestra F Chowdhary N. Maternal mental health matters: indicators for perinatal mental health—a scoping review. PLoS One. (2025) 20:e0317998. 10.1371/journal.pone.0317998 39869575 PMC11771939

[18] Trang DTH Ha BTT Vui LT Chi NTQ Thi LM Duong DTT. Understanding the barriers to integrating maternal and mental health at primary health care in Vietnam. Health Policy Plan. (2024) 39:541–51. 10.1093/heapol/czae027 38597872 PMC11145914

[19] Gab-deedam S Sampson S Wonodi C Atobatele S Ebinim H Ayodeji O. Landscape analysis of primary healthcare integration; understanding barriers and enablers for people-centered care in low-and-middle income countries. Int J Integr Care. (2023) 23:591. 10.5334/ijic.ICIC23224

[20] Serván-Mori E Cerecero-García D Hone T Castro A Garcia-Diaz R Millett C. Fragmented healthcare and effective maternal coverage in Mexico, 2009–2023. BMC Med. (2026) 24:74. 10.1186/s12916-025-04531-y PMC12882126 41514335

[21] Mor S Sela Y Lev-Ari S. Postpartum mothers’ mental health in a conflict-affected region: a cross-sectional study of emotion regulation and social support. J Clin Med. (2025) 14:1244. 10.3390/jcm14041244 40004775 PMC11856334

[22] Duberstein ZT Brunner J Panisch LS Bandyopadhyay S Irvine C Macri JA. The biopsychosocial model and perinatal health care: determinants of perinatal care in a community sample. Front Psychiatry. (2021) 12:746803. 10.3389/fpsyt.2021.746803 34867537 PMC8635705

[23] Bergunde L Garthus-Niegel S Alexander N Steudte-Schmiedgen S. Perinatal mental health research: towards an integrative biopsychosocial approach. J Reprod Infant Psychol. (2022) 40:325–8. 10.1080/02646838.2022.2101781 35861195

[24] Moyer SW Kinser PA. A comprehensive conceptual framework to guide clinical practice and research about mental health during the perinatal period. J Perinat Neonatal Nurs. (2021) 35:46–56. 10.1097/JPN.0000000000000535 33528187

[25] Manolova G Waqas A Chowdhary N Salisbury TT Dua T. Integrating perinatal mental healthcare into maternal and perinatal services in low and middle income countries. Br Med J. (2023) 381:e073343. 10.1136/bmj-2022-073343 PMC10203867 37220917

[26] Kirkbride JB Anglin DM Colman I Dykxhoorn J Jones PB Patalay P. The social determinants of mental health and disorder: evidence, prevention and recommendations. World Psychiatry. (2024) 23:58–90. 10.1002/wps.21160 PMC10786006 38214615

[27] Rubio-León DC Cano-Sierra L Reyes-Rivera M-J Abitbol P García-Padilla D Forero-García SL. Social determinants of mental health in armed conflict-affected municipalities. Psychosoc Interv. (2026) 35:746803. 10.5093/pi2026a1 PMC12826549 41583828

[28] Aldabbour B Elamassie S Mahdi S Abuzaid H Abed T Tannira Y. Exploring maternal and neonatal health in a conflict-affected setting: cross-sectional findings from Gaza. Confl Health. (2025) 19:45. 10.1186/s13031-025-00687-9 40676703 PMC12269202

[29] Seema M Afoakwah C Tuzel E Byrnes J. The impact of disasters on maternal mental health: a systematic review. J Glob Health. (2026) 16:04126. 10.7189/jogh.16.04126 41990306 PMC13086487

[30] Hassan GD Abshir JNL Osman F. Depression and anxiety symptoms among women in post-conflict Somalia: a cross-sectional study in maternal and child health centres. Front Glob Womens Health. (2026) 6:1652133. 10.3389/fgwh.2025.1652133 41788305 PMC12958141

[31] Prom MC Denduluri A Philpotts LL Rondon MB Borba CPC Gelaye B. A systematic review of interventions that integrate perinatal mental health care into routine maternal care in low- and middle-income countries. Front Psychiatry. (2022) 13:859341. 10.3389/fpsyt.2022.859341 35360136 PMC8964099

[32] Waqas A Koukab A Meraj H Dua T Chowdhary N Fatima B. Screening programs for common maternal mental health disorders among perinatal women: report of the systematic review of evidence. BMC Psychiatry. (2022) 22:54. 10.1186/s12888-022-03694-9 35073867 PMC8787899

[33] Agampodi T Amarasinghe G Wickramasinghe A Wickramasinghe N Warnasekara J Jayasinghe I. Incorporating early pregnancy mental health screening and management into routine maternal care: experience from the Rajarata Pregnancy Cohort (RaPCo), Sri Lanka. BMJ Glob Health. (2023) 8:e012852. 10.1136/bmjgh-2023-012852 37748792 PMC10533714

[34] McNab SE Dryer SL Fitzgerald L Gomez P Bhatti AM Kenyi E. The silent burden: a landscape analysis of common perinatal mental disorders in low- and middle-income countries. BMC Pregnancy Childbirth. (2022) 22:342. 10.1186/s12884-022-04589-z 35443652 PMC9019797

[35] Bou-Orm IR Moussallem M Karam J Delara M Varma V Diaconu K. Provision of mental health and psychosocial support services to health workers and community members in conflict-affected northwest Syria: a mixed-methods study. Confl Health. (2023) 17:46. 10.1186/s13031-023-00547-4 37794393 PMC10548701

[36] Tembo C Portsmouth L Burns S. Identification of mothers with mental health problems is accidental: perceptions of health care providers on availability, access, and support for maternal mental health care for adolescent mothers in Malawi. BMC Health Serv Res. (2024) 24:983. 10.1186/s12913-024-11469-z 39187823 PMC11346021

[37] ElKhalil R Menon P Adam H Bayoumi R Qurniyawati E Masuadi E. Professional, organizational and policy-level barriers and facilitators to perinatal mental health care in the United Arab Emirates: a qualitative study. PLoS One. (2026) 21:e0344312. 10.1371/journal.pone.0344312 41790859 PMC12965694

[38] Waqas A Zafar SW Meraj H Tariq M Naveed S Fatima B. Prevention of common mental disorders among women in the perinatal period: a critical mixed-methods review and meta-analysis. Global Mental Health. (2022) 9:157–72. 10.1017/gmh.2022.17 36618726 PMC9806961

[39] Troup J Fuhr D Woodward A Sondorp E Roberts B. Barriers and facilitators for scaling up mental health and psychosocial support interventions in low- and middle-income countries for populations affected by humanitarian crises: a systematic review. Int J Ment Health Syst. (2021) 15:5. 10.1186/s13033-020-00431-1 33413526 PMC7792016

